# Standardization of a spinal cord lesion model and neurologic evaluation using mice

**DOI:** 10.6061/clinics/2018/e293

**Published:** 2018-02-22

**Authors:** Paulo Alvim Borges, Alexandre Fogaça Cristante, Tarcísio Eloy Pessoa de Barros-Filho, Renato Jose Mendonça Natalino, Gustavo Bispo dos Santos, Raphael Marcus Marcon

**Affiliations:** Instituto de Ortopedia e Traumatologia (IOT), Hospital das Clinicas HCFMUSP, Faculdade de Medicina, Universidade de Sao Paulo, Sao Paulo, SP, BR

**Keywords:** Reference Standards, Mice, Spinal Cord, Spinal Cord Compression

## Abstract

**OBJECTIVE:**

To standardize a spinal cord lesion mouse model.

**METHODS:**

Thirty BALB/c mice were divided into five groups: four experimental groups and one control group (sham). The experimental groups were subjected to spinal cord lesion by a weight drop from different heights after laminectomy whereas the sham group only underwent laminectomy. Mice were observed for six weeks, and functional behavior scales were applied. The mice were then euthanized, and histological investigations were performed to confirm and score spinal cord lesion. The findings were evaluated to prove whether the method of administering spinal cord lesion was effective and different among the groups. Additionally, we correlated the results of the functional scales with the results from the histology evaluations to identify which scale is more reliable.

**RESULTS:**

One mouse presented autophagia, and six mice died during the experiment. Because four of the mice that died were in Group 5, Group 5 was excluded from the study. All the functional scales assessed proved to be significantly different from each other, and mice presented functional evolution during the experiment. Spinal cord lesion was confirmed by histology, and the results showed a high correlation between the Basso, Beattie, Bresnahan Locomotor Rating Scale and the Basso Mouse Scale. The mouse function scale showed a moderate to high correlation with the histological findings, and the horizontal ladder test had a high correlation with neurologic degeneration but no correlation with the other histological parameters evaluated.

**CONCLUSION:**

This spinal cord lesion mouse model proved to be effective and reliable with exception of lesions caused by a 10-g drop from 50 mm, which resulted in unacceptable mortality. The Basso, Beattie, Bresnahan Locomotor Rating Scale and Basso Mouse Scale are the most reliable functional assessments, and but the horizontal ladder test is not recommended.

## INTRODUCTION

Traumatic spinal cord injury (SCI) is a medical challenge. SCI causes great damage to patients 1,2; to date, no effective treatment for SCI has been described 3,4.

The study of the physiopathology of these lesions provides evidence regarding different treatment strategies and contributes to progress toward overcoming this challenge. Several study models have been tested, and animal models remain the most frequently used. Rodent models are the most common type of animal model 5,6. Between 2004 and 2014, out of 407 studies, 289 investigations (71%) used rats, and 69 investigations (16.9%) used mice 7.

Mouse models have advantages compared with other animal models. Mice are relatively inexpensive for laboratories, are easy to breed, have a high reproductive rate, require little care, are easy to manipulate, and have tremendous potential for genetic engineering 8,9.

The standardization of a study model allows for the appropriate reproduction of SCI and the analysis of research results 10. For this reason, to continue the search for effective treatments, this study had the objective of standardizing a mouse model of SCI in a laboratory with experience with SCI in rats.

## MATERIALS AND METHODS

Thirty BALB/c mice between 7 and 9 weeks of age and between 20 and 40 grams were randomly selected. This sample size was based on prior studies 11,12,13. No animals with clinically visible diseases or motricity abnormalities were accepted. Our research laboratory strictly adheres to all international guidelines regarding handling and pain control in the care and use of animals in research. Three animals were housed in each cage in the laboratory and were handled and stimulated to move prior to the experiment such that they could become accustomed to the researchers and to the motor function evaluation experiment conducted after SCI. Food and water were provided ad libitum for the duration of the study.

The specimens were divided into five groups of six mice:

Group 1: Control group (“sham”) – laminectomy only.
Group 2: SCI by weight drop (10 g) from a height of 6.25 mm.
Group 3: SCI by weight drop (10 g) from a height of 12.5 mm.
Group 4: SCI by weight drop (10 g) from a height of 25 mm.
Group 5: SCI by weight drop (10 g) from a height of 50 mm.
Randomization and blinding occurred at each step of the experiment. Animals were weighed before and after the experiment to confirm that they received good housing and care.

Animals that presented with autophagia or without an appreciable lesion after the experiment were excluded. The surgical procedure was completed under general anesthesia as described by Gargiulo (2012). A dorsal approach was used to expose the posterior aspect of the T7 to T11 vertebrae. Every step of the surgery was conducted with microscope visualization. Eventually, a T9 laminectomy was performed, and animals were prepared for SCI.

**Figure 1A and 1B:**
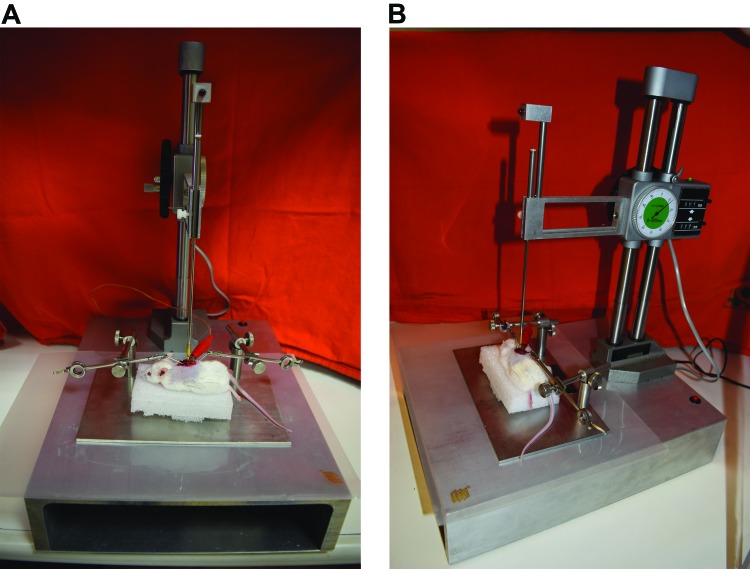
Mouse prepared for SCI in an NYU Impactor device (as seen from different angles).

SCI was induced in accordance with the MASCIS (*Multicenter Animal Spinal Cord Injury Study*) protocol 15 using a New York University impactor (NYU Impactor) 16 as shown in Figures 1A and 1B.

The lesion parameters were set as stated above in the description of the experimental characteristics. Each animal was positioned under the impactor apparatus. The lesion was caused by the impact of a 10-g rod in free fall from directly over the exposed spinal cord. Fall height was correlated with lesion severity. After SCI was induced, the wound was closed, and the animals were observed and cared for six weeks. Once the possibility of urinary retention caused by neurogenic bladder was detected, vesical massage was performed daily for every animal. All complications were recorded (including infection, autophagia, corporal weight loss and death).

For six weeks, the animals were tested using the following behavioral scales: 1) the Basso, Beattie, Bresnahan Locomotor Rating Scale (BBB) 17; 2) the Basso Mouse Scale (BMS) 18; 3) the Mouse Function Scale (MFS) 19; and 4) the horizontal ladder test 20.

At the end of six weeks, the animals were anesthetized again and were then euthanized by cardiac perforation. The spine was removed *en bloc* and sent for histological analysis. Two representative fields from the spinal cord with a thickness of 200 μm that were 1 mm distal and 1 mm proximal to the center of the lesion were prepared and stained with hematoxylin and eosin. The spine was cut in the sagittal plane. A single, experienced pathologist, blinded to the allocation of the mice, performed analyses and documented the absence (grade 0) or presence (grade 1, 2 or 3) of hyperemia, degeneration, and cellular infiltrate. Grades were determined based on the extent to which the spinal cord presented these pathological findings. Grade 1 indicated the presence of such findings in up to one-third of the spinal cord width in the lesion area, grade 2 indicated such findings in one to two-thirds of the spinal cord width, and grade 3 indicated such findings in more than two-thirds of the spinal cord width. The average score of the two fields is the final score.

Descriptive statistics are expressed as the means and standard deviations and were tested for normality using the Kolmogorov-Smirnov test. Normally distributed data were assessed using parametric tests. The Kruskal-Wallis test was used to analyze the functional scale results. For comparisons between weeks in each group, the Friedman test was used because the data were not normally distributed (*p*<0.05). Spearman’s test was used to evaluate correlations between groups, histology findings and functional scale results. We started from the null hypothesis and considered a probability of an alpha error of 5%. We used Statistical Package for Social Sciences (SPSS) v23.0 for Mac (IBM Corp., Armonk, NY) for our analyses.

### Ethics

This study was approved by the scientific board of the Orthopedic Department of the Clinics Hospital of São Paulo University (Hospital das Clinicas da Universidade de São Paulo) and by the Animal Experimentation Ethics Committee of the School of Medicine of São Paulo College. We followed guidelines established by the Canadian Council on Animal Care (CCAC), the Brazilian School of Animal Experimentation (Colégio Brasileiro de Experimentação Animal - COBEA) and the National Council for Control of Animal Experimentation (Conselho Nacional de Controle de Experimentação Animal - CONCEA).

## RESULTS

Thirty mice were subjected to experimentation with six mice per group. Three mice died of urinary infection within 10 days after the experiment, including one mouse in Group 3, one mouse in Group 4 and one mouse in Group 5. Another three mice in Group 5 also died, including two that did not survive the experiment and one that died six hours after the experiment. One mouse in Group 4 developed autophagia and was excluded from the experiment on the 13^th^ day. No mice presented with weight loss during the experiment, confirming that the animals received adequate care. Group 5 was excluded from the statistical analysis due to data loss.

**Figure 2:**
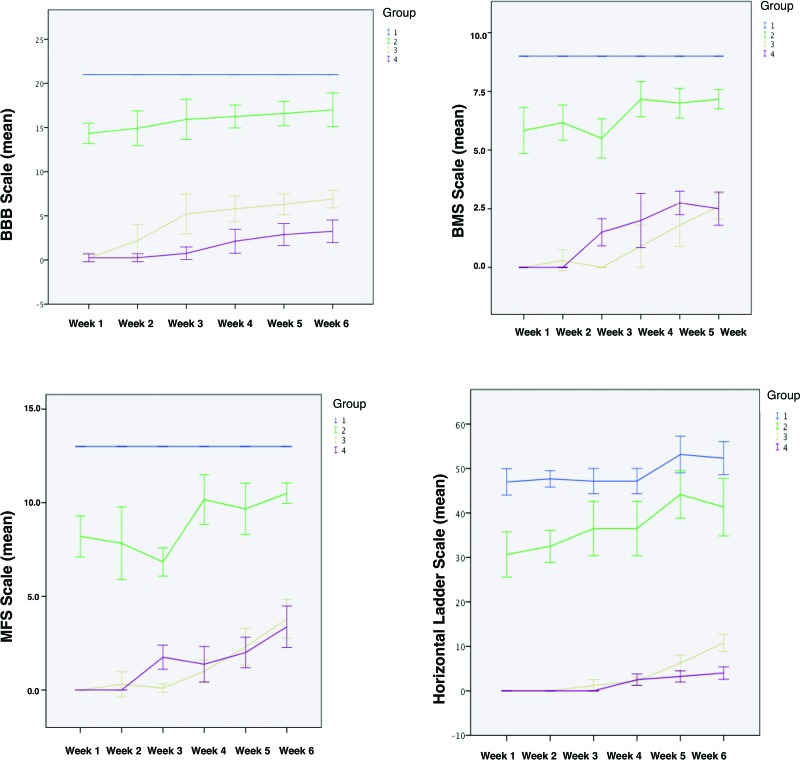
Mean results of the functional scales during each week after SCI induction.

Statistically significant results were obtained for all functional scales (the BBB, BMS, MFS and horizontal ladder test); in particular, for both the Friedman and Kruskal-Wallis tests, *p*<0.05 was observed among all the groups and all weeks of the experiment. These findings demonstrated that the groups were significantly different and changed over the course of the experiment (i.e., motor function evolved significantly over time). Worse SCI was associated with worse functional scores, with the worst and best results obtained for Group 4 and Group 1, respectively. Each group exhibited differences over time on all tested scales. For each group, comparisons over time revealed a functional recovery pattern that was consistent across all scales. This pattern consisted of the progressive recovery of functional scores. A more severe lesion corresponded to a slower and more limited recovery. The results of the functional scales are shown in Figure 2.

Similarly, histology analysis produced results that were correlated with the severity of SCI among the different experimental groups. More severe lesions were associated with worse histological grades. The histology results are summarized in Table 1.

**Figure 3:**
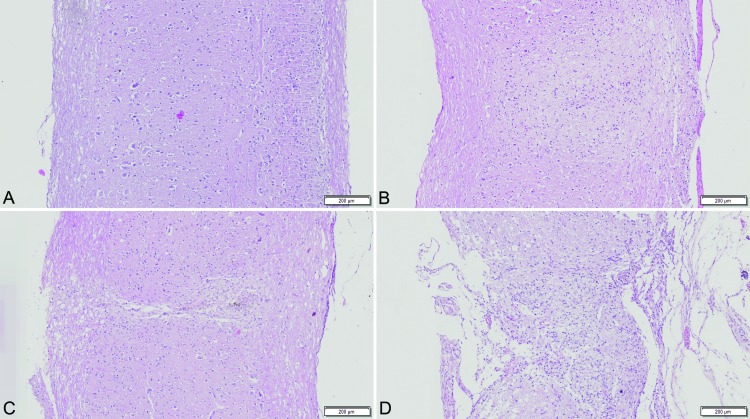
Histological sections of the spinal cord (200 mm thick; hematoxylin-eosin staining). “A” is a histological section of a normal uninjured spinal cord. “B” shows mild SCI, “C” shows moderate SCI, and finally “D” shows severe SCI with complete unstructured architecture of the spinal cord.

SCI is demonstrated in the histological sections shown in Figure 3.

Spearman’s test revealed correlations between the functional scale results and most of the histological results; however, the horizontal ladder test findings were not associated with either hyperemia or cellular infiltrate results. Spearman’s correlation results are summarized in Table 2.

## DISCUSSION

The lack of effective therapy for SCI and its high morbidity and mortality justify continued research regarding this topic.

The objective of our study was to standardize a traumatic SCI model in a laboratory with extensive experience using rats for SCI experimentation. The rationale was to continue research relatively inexpensively and with high genetic engineering potential; both objectives can be achieved using mouse models.

We chose BALB/c mice because they are readily available at our institution. The specimens examined in this study were young adults of average weight. Of the thirty mice used in our study, 6 mice died. This data loss of 20%, which would potentially compromise this study, produced a clear bias in our evaluations. A major factor in this high rate of data loss was the single group that accounted for 66.67% of the total deaths. The exclusion of this group resulted in a mortality rate of 2/24 mice (8.33%). We believe that the Group 5 treatment produced a severe, nonviable SCI that is not usable as a model. The other groups had an acceptable mortality rate and could be reasonably assessed. Unfortunately, since every mouse within a group was subjected to experimentation on the same day, it was not possible to limit deaths before the end of the experiment (three mice died within six hours of the SCI procedure). Additional mice were not subjected to the severity of treatment experienced by Group 5 mice because we concluded that it would be unethical to expose animals to such a high mortality risk solely to increase the statistical power of our results. Autophagia is a common complication in rodent SCI models but presented at a very low rate in our study (1/30 mice).

Functional scales are frequently used in animal SCI models and are believed to be the best clinical outcome measures for such models 21. Histological analyses constitute another frequently used outcome measure; histological findings provide objective proof of SCI and its grade and serve as a control for functional scale comparisons.

The analysis of the results of the functional scales in this study demonstrated that, as expected, the groups were different among themselves, and the SCI was more severe as the height of the weight dropped increased. These results make sense; the higher the drop, the greater the impact and trauma and thus, the worse the SCI should be. Upon in-depth evaluation of the results shown in Figure 2, we see that Groups 3 and 4 present similar functional scores and have greater differences compared with Groups 1 and 2. These results were also expected, as mice in Groups 3 and 4 were subjected to the most severe SCI. The data in Figure 2 also show that the mice begin to demonstrate functional improvement on the BBB scale earlier than on the other scales (improvement on the BBB scale starts at week 2; improvement on the other scales starts at week 3). This observation suggests that the BBB scale has greater sensitivity than the other scales studied. Interestingly, we subjectively felt that the BBB was harder to implement and requires more time. Those outcomes were not evaluated because they were not in the original study plan. Progressive recovery was demonstrated in all the scales assessed. As expected, groups with more severe lesions (Groups 3 and 4) had poorer final outcomes compared with the groups with less severe lesions.

In our study, the presence of SCI was confirmed by histology. The histological parameters evaluated were those that could be assessed with sections stained for hematoxylin-eosin because we did not have access to other stains. As shown in Table 1, as the SCI severity increases, the presence and severity of the histological findings also increase. As expected, more severe lesions present more histological abnormalities. Hyperemia, degeneration and cellular infiltrate are all histological findings of late SCI 22,23 and are thus appropriate in our study, as the animals were sacrificed 6 weeks after SCI. Additionally, we did not find histological changes indicative of early SCI, such as necrosis or hemorrhage, in the sections assessed. As shown in Figure 3, we could clearly identify four patterns of SCI with different severities. As expected, increased severity corresponded to an increase in disorganization of the cellular architecture.

Correlations between the scale-based and histological findings were calculated and were strong in most cases (as shown in Table 2), confirming the reliability of the established model. Among the tested scales, only the BBB and BMS scales showed strong correlations with all the histological results; thus, in our opinion, these scales were the most appropriate scales for our model. Although we did not attempt to quantify the ease of application of the tested scales in mice, we subjectively believe that the BMS scale felt relatively quick and straightforward to use, and we recommend this scale for future studies. In contrast, the horizontal ladder test results were weakly correlated with cellular infiltrate and were not correlated with hyperemia. Although these findings do not fully invalidate the use of this test because there was a strong correlation with neuronal degeneration, we do not recommend the further use of the horizontal ladder test because better scales have been thoroughly validated in the current study.

Our study has a number of limitations. High mortality is a strongly negative aspect of the study. All the animals were subjected to SCI on the same day; because most of the animals that died did so within hours after SCI, the excessive mortality was unfortunately unavoidable. In the future, studies experimenting with fewer animals at a time is advisable to prevent excessive mortality. Also, the study could not use alternative histological stains as other studies had done previously; the use of different stains could better evaluate damage to the myelin sheaths and neurons after SCI 11,12,13. Other SCI evaluation methods such as evoked motor potential could not be applied in this study but could provide more information about the nature of the SCI in this model. Our laboratory anticipates future studies using different evaluation methods to better assess this SCI mouse model.

On the other hand, the study has a number of advantages. Although there was high mortality, the results showed solid statistical significance with a relatively low overall number of mice. Additionally, we established a limit of acceptable SCI severity in mice (above which excessive mortality occurs), which is highly useful and has not been reported in other studies. Our histology method is also advantageous. Although it might appear incomplete given its simplicity, the hematoxylin-eosin staining managed to adequately correlate the clinical and histological findings with a simpler, less expensive approach that uses readily available histological resources when compared to the staining approaches used in other studies. Finally, our study might be one of the few to use the horizontal ladder test to evaluate functional motor in mice subjected to SCI.

This study effectively demonstrated a simple, reliable, inexpensive and reproducible mouse model of SCI and as well as presented adequate neurological evaluations. This standardization can be used in future studies of SCI that use mouse models.

Our mouse model of SCI is effective, reproducible and reliable with the exception of SCI induced by a 10-g weight drop from a height of 50 mm, which produces unacceptable mortality rates. Among the functional scales that were used, the BBB and BMS scales are the most reliable, but the horizontal ladder test is not recommended.

## AUTHOR CONTRIBUTIONS

Borges PA designed the study, interpreted the results, wrote the manuscript and approved the final version to be published. Cristante AF designed the study, interpreted the results, wrote the manuscript and approved the final version to be published. Barros-Filho TE designed the study, interpreted the results, wrote the manuscript and approved the final version to be published. Natalino RJ collected data and interpreted results (histology). dos Santos GB designed the study and collected data (animal experimentation). Marcon RM designed the study, interpreted the results, wrote the manuscript and approved the final version to be published.

## Figures and Tables

**Table 1:** Histology results.

		HYPEREMIA	DEGENERATION	INFILTRATE
				
**Group 1**	**Mouse 1**	0	0	0
	**Mouse 2**	0	0	0
	**Mouse 3**	0	0	0
	**Mouse 4**	0	1	0
	**Mouse 5**	0	0	0
	**Mouse 6**	0	0	0
**Group 2**	**Mouse 1**	0	0	0
	**Mouse 2**	1	2	2
	**Mouse 3**	0	2	1
	**Mouse 4**	1	0	0
	**Mouse 5**	1	1	1
	**Mouse 6**	2	1	1
**Group 3**	**Mouse 1**	2	2	1
	**Mouse 2**	2	3	1
	**Mouse 3**	1	1	0
	**Mouse 4**	2	2	2
	**Mouse 6**	1	2	2
**Group 4**	**Mouse 1**	0	2	2
	**Mouse 3**	2	3	2
	**Mouse 4**	3	3	3
	**Mouse 5**	2	3	2

**Table 2:** Spearman’s correlation test between the histology results and the functional scales.

Parameter/Scale	BBB	BMS	MFS	Horizontal Ladder
**Degeneration**	ρ = -0.813	ρ = -0.794	ρ = -0.696	ρ = -0.670
**Cellular Infiltrate**	ρ = -0.776	ρ = -0.828	ρ = -0.760	ρ = -0.238
**Hyperemia**	ρ = -0.773	ρ = -0.789	ρ = -0.678	ρ = 0.077
